# Highlights of the 2018 Chinese hypertension guidelines

**DOI:** 10.1186/s40885-020-00141-3

**Published:** 2020-05-01

**Authors:** Jing Liu

**Affiliations:** Department of Cardiology, Peking University People’s Hospital, Beijing, 100044 China

**Keywords:** Chinese, Hypertension, Guidelines

## Abstract

**Background:**

Blood pressure (BP) are uncontrolled in over 80% hypertensive population in China, indicating a compelling need for a pragmatic hypertension management strategy. The 2018 Chinese hypertension guidelines issued in 2019, after 3 years revision. During the periods, the latest United States (US) and European guidelines successively published, bringing new thoughts, wisdoms and schemes on hypertension management. This review aims to summarize the highlights of the new Chinese guidelines.

**Main text:**

Despite the fact that the 2017 US hypertension guidelines changed hypertension definition from ≥140/90 mmHg to 130/80 mmHg, the Chinese hypertension guidelines did not follow suit, and maintained 140/90 mmHg as the cut-point of for diagnosis of hypertension. A combined, cardiovascular risks and BP levels-based antihypertensive treatment algorithm was introduced. Five classes of antihypertensive drugs, including β-blockers were recommended as initiation and maintenance of BP-lowering therapy. Initiating combination therapy, including single pill combination (SPC) was indicated in high-risk patients or those with grade 2 or 3 hypertension. For those with grade 1 hypertension (BP ≥ 140/90 mmHg), an initial low-dose antihypertensive drugs combination treatment could be considered.

**Conclusions:**

China has never stopped exploring the best strategy for improving hypertension control. Based on clinical evidence and expertise, the newest Chinese guidelines and expert consensus will be of help in guiding physicians and practitioners to provide better management of hypertension in China.

## Background

After 3 years and more than 30 symposiums on revision, the final English version of the 2018 Chinese Guidelines for Prevention and Treatment of Hypertension (Revised Edition) was officially published in March, 2019 [1]. During these periods, the United States (US) and Europe successively updated the hypertension guidelines [2, 3]. The 2018 Chinese hypertension guidelines revision committee borrowed experience from colleagues of the international societies, combined with expertise in clinical practice and evidence accumulated from population studies and clinical trials on blood pressure (BP)-lowering therapy in China and worldwide, and formed Chinese characteristic guidelines for management of hypertension.

Hypertension is the leading risk factors of mortality in China, accounted for 2·54 million deaths in 2017 and most were due to cardiovascular diseases [4]. It was estimated about 23.2% (244.5 million) Chinese adults had hypertension, the awareness and treatment rate are 46.9 and 40.7% respectively, and only 15.3% was controlled [5]. Over 20 years, Chinese Hypertension League (CHL) has issued 4 editions of national hypertension guidelines since 1999, with the endorsement of Chinese Society of Cardiology (CSC) and other organizations. In past, the former three editions have played important role in guiding clinical practice and improving hypertension management. The 2018 Chinese hypertension guidelines will provide millions of doctors in China with a clear and accessible roadmap for hypertension control.

What are the key points of the 2018 Chinese hypertension guidelines?

## Main text

### Blood pressure measurement

The 2018 Chinese hypertension guidelines highlight that accurate BP measurement is the fundamental for assessing BP levels, establishing diagnosis of hypertension and evaluating the efficacy of antihypertensive treatment.

Clinic (Office) BP measurement (CBPM) currently remains the common method measuring BP in outpatient clinics in China. Meanwhile out-of-office BP measurements, including ambulatory BP monitoring (ABPM) or home BP monitoring (HBPM), are advocated to confirm the diagnosis of hypertension, identify white-coat hypertension or masked hypertension, evaluate BP variation, and assess antihypertensive efficacy, if available. In addition, HBPM can improve the adherence of antihypertensive treatment, and might be of help improving BP control. With the development of telemetry technology and equipment, internet-based BP remote monitoring is expected to become a new model for BP management in the future.

### Definition and classification of hypertension

2017 US hypertension guidelines changed the definition of hypertension from the general accepted cut-point of 140/90 mmHg to 130/80 mmHg [2], arousing controversy worldwide.

Given that the awareness, treatment and control rate of hypertension in China are still low, changing threshold for diagnosis of hypertension to ≥130/80 mmHg will dramatically increase the volume of hypertensive patients, including those need to treat, which will inevitably bring huge disease burden and much more medical expenditure [6]. On the other hand, changing hypertension definition to ≥130/80 mmHg is not endorsed by most of international hypertension societies, the later released 2018 European hypertension guidelines refused to make any alternation on this issue. Therefore, the 2018 Chinese hypertension guidelines did not follow suit with the US guidelines and maintained the current cut-point of ≥140/90 mmHg for hypertension diagnosis. The latest issued Korean and Japanese hypertension guidelines also maintained 140/90 mmHg as the definition criteria of hypertension [7–10].

BP classification and comparison between Chinese and international hypertension guidelines could be seen as following (Table 1).

**Table 1:** BP categories in Chinese, Korean, Japanese, US and European hypertension guidelines

BP category (mmHg)	CHL 2018 [1]	KSH 2018 [7–9]	JSH 2019 [10]	AHA/ACC 2017 [2]	ESC/ESH 2018 [3]
SBP < 120 and DBP < 80	Normal	Normal	Normal	Normal	Optimal
SBP: 120–129 and DBP < 80	High normal	Elevated	High normal	Elevated	Normal^a^
SBP: 130–139 and (or) DBP: 80–89		Prehypertension	Elevated	Grade 1 hypertension	High normal^b^
SBP: 140–159 and (or) DBP: 90–99	Grade 1 hypertension	Grade 1 hypertension	Grade 1 hypertension	Grade 2 hypertension	Grade 1 hypertension
SBP: 160–179 and (or) DBP: 100–109	Grade 2 hypertension	Grade 2 hypertension	Grade 2 hypertension		Grade 2 hypertension
SBP ≥ 180 and (or) DBP ≥ 110	Grade 3 hypertension		Grade 3 hypertension		Grade 3 hypertension
SBP ≥ 140 and DBP < 90	ISH	ISH	ISH	NA	ISH

*ACC* American College of Cardiology, *AHA* American Heart Association, *BP* blood pressure, *CHL* Chinese Hypertension League, *DBP* diastolic BP, *ESC* European Society of Cardiology, *ESH* European Society of Hypertension, *ISH* isolated systolic hypertension, *JSH* Japanese Society of Hypertension, *KSH* Korean Society of Hypertension, *NA* not available, *SBP* systolic BP.

^a^ DBP: 80–84 mmHg

^b^ DBP: 85–89 mmHg.

### Cardiovascular risk stratification

Cardiovascular (CV) risk stratification in hypertensive patients is the basis of initiating antihypertensive therapy and the determinants of establishing appropriate BP targets and antihypertensive treatment strategies.

In the 2018 Chinese hypertension guidelines, hypertensive patients are classified to one of the following CV risk strata, from low risk, moderate risk, high risk to very high risk (Table 2), referring to the BP levels and CV risk factors, target organ damages or complications. Similar with 2018 European hypertension guidelines [3], BP range between 130 and 139/85–89 mmHg is also added in the category for CV risk evaluation.

**Table 2:**
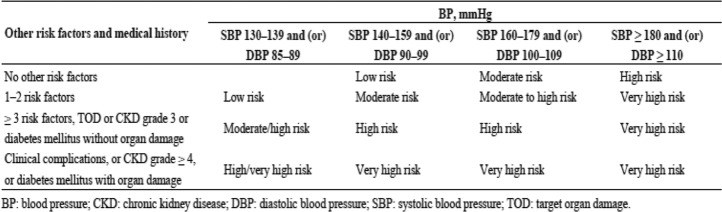
Cardiovascular risk stratification in patients with elevated BP

In addition to the traditional CV risk factors, hyperhomocysteinemia is recognized as a meaningful predictor for stroke, a highly prevalent complication of hypertension in Chinese population, based on the evidence from cohort study and Chinese Stroke Primary Prevention Trial (CSPPT) [11, 12], with a modified cut-point of ≥15umol/L (Table 3). This is unique, and not seen in the Korean and Japanese hypertension guidelines [7–10].

**Table 3:** Factors influencing cardiovascular prognosis in hypertensive patients

Cardiovascular risk factors	TOD	Concomitant clinical diseases
● Hypertension (Grade 1–3) ● Man > 55 years ● Woman > 65 years ● Smoking or passive smoking ● Impaired glucose tolerance (7.8–11.0 mmol/L for 2-h blood glucose) and/or impaired fasting glucose (6.1–6.9 mmol/L) ● Dyslipidemia TC ≥ 5.2 mmol/L (200 mg/dL) or LDL-C ≥ 3.4 mmol/L (130 mg/dL) or HDL-C < 1.0 mmol/L (40 mg/dL) ● Family history of early onset cardiovascular disease (onset of first-degree relatives at age < 50 years) ● Abdominal obesity (waist circumference: Man ≥90 cm, Woman ≥85 cm) or obesity (BMI ≥ 28 kg/m2) ●Hyperhomocysteinemia (> = 15umol/L)	● Left ventricular hypertrophy electrocardiogram: Sokolow-Lyon voltage > 3.8 mV or Cornell product > 244 mV·ms Echocardiogram: LVMI (man ≥115 g/m2 woman ≥95 g/m2) ● Carotid ultrasonography (IMT ≥ 0.9 mm) or atherosclerotic plaque ● Carotid-femoral pulse wave velocity ≥ 12 m/s (*optional) ● Ankle/Brachial index < 0.9 (*optional) ● Reduced estimated glomerular filtration rate (eGFR 30–59 mL/min per 1.73 m2) or slight increase in serum creatinine: Man 115–133 mol/L (1. 3–1. 5 mg/dL), Woman 107–124 mol/L (1.2–1.4 mg/dL) ●Microalbuminuria: 30–300 mg/24 h or albumin/creatinine ratio ≥ 30 mg/g (3.5 mg/mmol)	● Cerebrovascular disease Cerebral hemorrhage Ischemic stroke Transient ischemic attack ● Heart disease History of myocardial infarction Angina pectoris Coronary revascularization Congestive heart failure Atrial fibrillation ● Renal disease: Diabetic nephropathy Renal dysfunction Including eGFR < 30 mL/min*1.73 m^2^; elevated serum creatinine: man ≥133 umol/L (1.5 mg/dL), woman ≥124 umol/L (1.4 mg/dL); proteinuria: (≥ 300 mg/24 h) ● Peripheral vascular disease ● Advanced retinopathy: Hemorrhages or exudates Papilloedema ● Diabetes mellitus Newly diagnosed: Fasting blood glucose ≥7.0 mmol/L (126 mg/dL); postprandial blood glucose ≥11.1 mmol/L (200 mg/dL) Treated but not controlled: Glycated hemoglobin: (HbA1c) ≥ 6.5%

*BMI* body mass index, *eGFR* estimated glomerular filtration rate, *HDL-C* high-density lipoprotein, *IMT* intima media thickness, *LDL-C* low-density lipoprotein; *LVMI* left ventricular mass index, *TC* total cholesterol, *TOD* Target organ damage.

### Treatment of hypertension

The 2018 Chinese hypertension guidelines recommend a treatment target of office BP (OBP) < 140/90 mmHg in general hypertensive patients, and further < 130/80 mmHg, if tolerated or in high-risk category. In older patients (65–79 years), it is recommended that systolic BP should be targeted to < 150 mmHg, and further < 140 mmHg, if tolerated. In elderly patients aged 80 years or over, a systolic BP target of < 150 mmHg is recommended.

BP targets in special population, such as diabetes mellitus (DM), chronic kidney disease (CKD) and post-stroke secondary prevention are also recommended in guidelines. A comparison of BP targets in Chinese and international guidelines could be seen as following (Table 4).

**Table 4:** BP targets in Chinese, Korean, Japanese, US and European hypertension guidelines

	CHL 2018 [1]	KSH 2018 [7–9]	JSH 2019 [10]	AHA/ACC 2017 [2]	ESC/ESH 2018 [3]
Young & middle-aged adults	< 140/90 mmHg^a^	< 140/90 mmHg^c^	< 130/80 mmHg	< 130/80 mmHg	120–130/70–79 mmHg
Elderly	65-79y < 150/90 mmHg^b^	≥65y < 140/90 mmHg	≥75y < 140/90 mmHg	≥65y < 130/80 mmHg	65–79y 130–139/ 70–79 mmHg
	≥80y < 150/90 mmHg				≥80y 130–139/ 70–79 mmHg
DM	< 130/80 mmHg	< 140/85 mmHg ^c^	< 130/80 mmHg	< 130/80 mmHg	120–130/70–79 mmHg^f^
CKD without proteinuria	< 140/90 mmHg	< 140/90 mmHg	< 140/90 mmHg	< 130/80 mmHg	130–139/70–79 mmHg
CKD with proteinuria	< 130/80 mmHg	< 130/80 mmHg	< 130/80 mmHg	< 130/80 mmHg	130–139/70–79 mmHg
Secondary prevention of stroke	< 140/90 mmHg	< 140/90 mmHg ^d^	< 130/80 mmHg ^e^	< 130/80 mmHg	120–130/70–79 mmHg^f^
CAD	< 140/90 mmHg^a^	< 130/80 mmHg	< 130/80 mmHg	< 130/80 mmHg	120–130/70–79 mmHg^f^
HFrEF	< 130/80 mmHg	< 130/80 mmHg	NA	< 130/80 mmHg	NA

*ACC* American College of Cardiology, *AHA* American Heart Association, *BP* blood pressure, *CAD* coronary artery disease, *CHL* Chinese Hypertension League, *CKD* chronic kidney disease, *DM* diabetes mellitus, *ESC* European Society of Cardiology, *ESH* European Society of Hypertension, *HFrEF* heart failure with reduced ejection fraction, *JSH* Japanese Society of Hypertension, *KSH* Korean Society of Hypertension, *NA* not available.

^a^ < 130/80 mmHg, if tolerable or high risk

^b^ < 140/90 mmHg, if tolerable

^c^ < 130/80 mmHg, if high risk.

^d^ < 130/80 mmHg, with lacunar infarction.

^e^ < 140/90 mmHg for bilateral cervical arteries stenosis, main cerebral artery occlusion or unevaluated.

^f^130–139/70–79 mmHg for people aged ≥65 years.

**Fig. 1:**
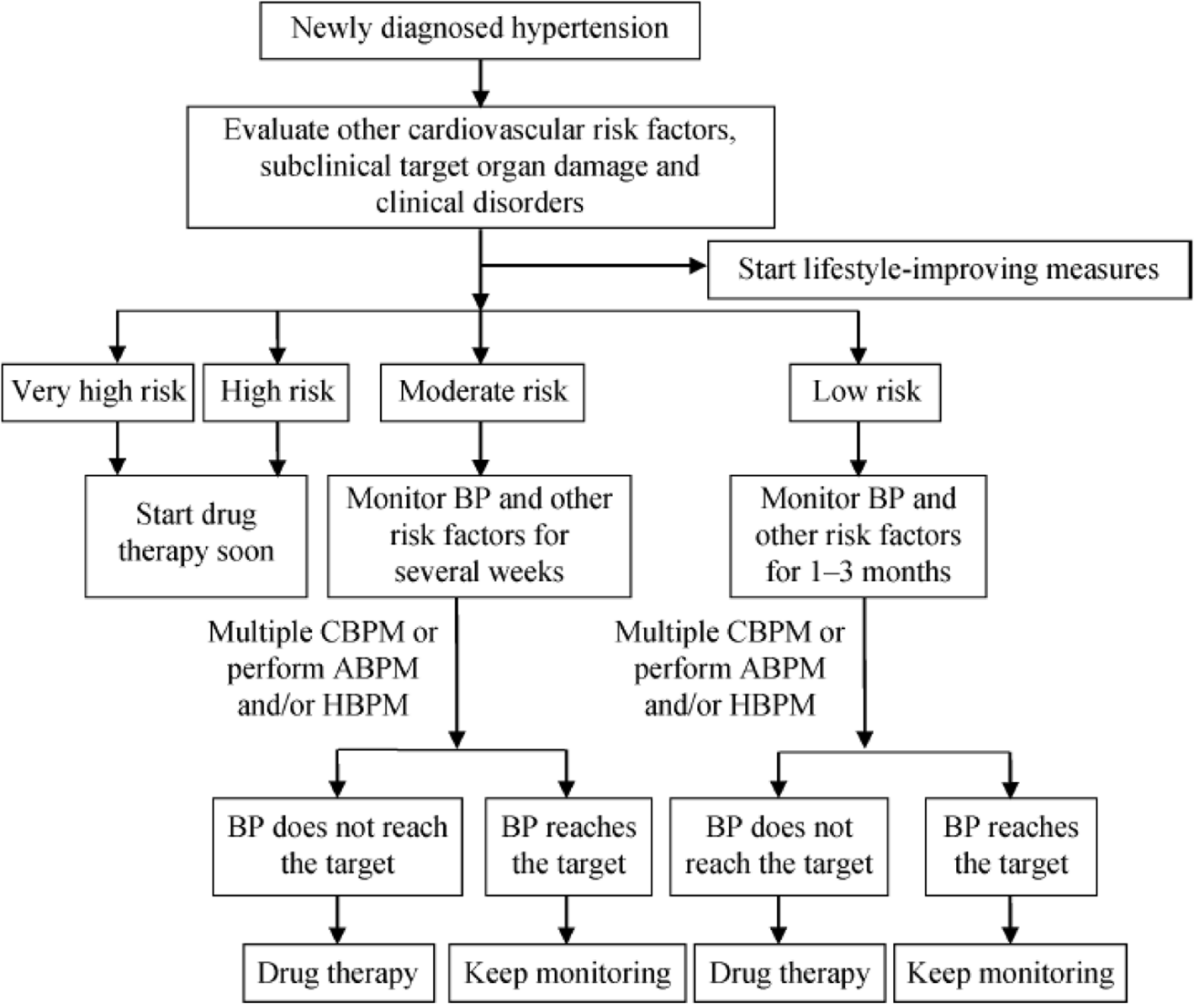
Evaluation and monitoring procedures for newly diagnosed hypertension. Diagnostic criteria of hypertension for ABPM: daytime mean SBP ≥ 135 mmHg or DBP ≥ 85 mmHg, nighttime mean SBP ≥ 120 mmHg or DBP ≥ 70 mmHg, or 24-h mean SBP ≥ 130 mmHg or DBP ≥ 80 mmHg; Criteria for HBPM: mean SBP ≥ 135 mmHg or DBP ≥ 85 mmHg. High risk patients with BP 130–139/85–89 mmHg or above, or moderate risk patients with BP ≥ 160/100 mmHg should start drug therapy immediately. ABPM: ambulatory blood pressure monitoring; BP: blood pressure; CBPM: clinic blood pressure measurement; DBP: diastolic blood pressure; HBPM: home blood pressure monitoring; SBP: systolic blood pressure

Antihypertensive treatment should be initiated on basis of CV risk assessment, combined with the BP levels (Fig. 1). Briefly, Patients with high or very high CV risk should initiate antihypertensive drug therapy immediately. Patients with low to moderate CV risk should also start antihypertensive agent treatment after several weeks’ lifestyle modification, alongside monitoring and follow-up.

Unlike 2017 US guidelines of kicking β-blockers off the first-line of antihypertensive therapy [2], the 2018 Chinese hypertension guidelines insist on recommending that all five classes of antihypertensive drugs, including calcium channel blockers (CCBs), angiotensin-converting enzyme inhibitors (ACEIs), angiotensin II receptor blockers (ARBs), diuretics and β-blockers, are suitable for the initiation and maintenance of BP-lowering therapy. This recommendation is in line with 2018 European hypertension guidelines and later published 2018 Korean guidelines [3, 7–9], while 2019 Japanese guidelines is in step with the US guidelines and recommend ACEIs、ARBs、CCBs and diuretics as the first-line antihypertensive agents in patients without compelling indications [10].

**Fig. 2:**
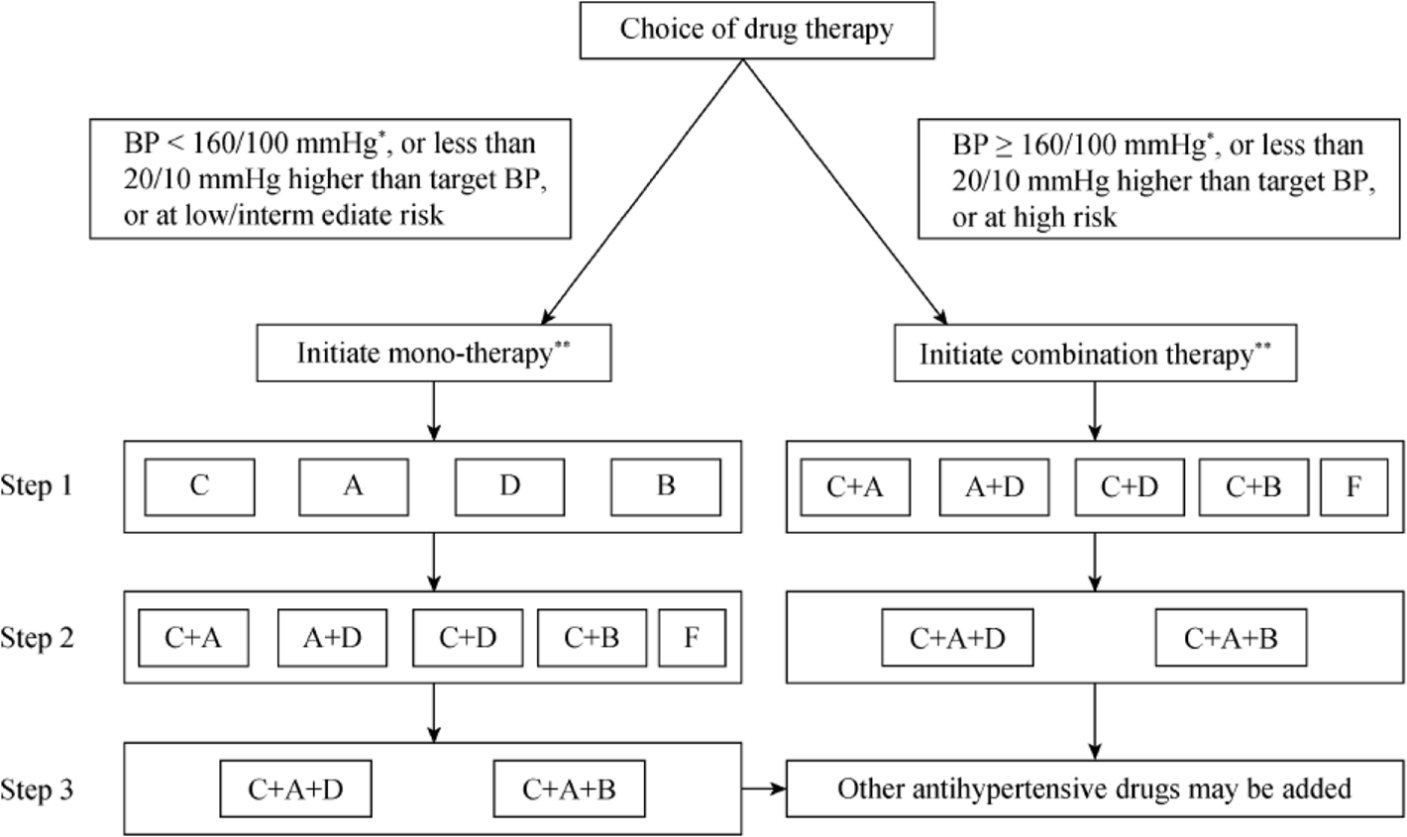
Flowchart for BP-lowering drugs therapy. A: ACEI or ARB; B: β-blockers; C:dihydropyridines CCB; D: thiazide-type diuretics; F: fixed-dose combination drugs.*For those with BP ≥ 140/90 mmHg and at high risk, initial low-dose combination therapy can also be recommended; **Including dosage titration and sequential addition of other agents to achieve BP target

Initiating combination therapy, including single pill combination (SPC) is indicated in high-risk hypertensive patients or those BP ≥160/100 mmHg or 20/10 mmHg above the BP target. For those BP ≥ 140/90 mmHg, an initial low-dose antihypertensive drugs combination therapy could also be considered. This is a more aggressive recommendation than ever, as the evidence from Systolic Pressure Intervention Trial (SPRINT) and meta-analysis uniformly demonstrated lower BP is better [13, 14]. If BP target not achieved, the dosage may be increased or combined with other antihypertensive agents. The 2018 Chinese hypertension guidelines provided a more flexible combination protocol, ACEI/ARB + CCB or diuretic, CCB + diuretic or CCB + β-blocker were all preferred combination options. (Fig. 2).

Of note, lifestyle interventions are addressed at all stage of hypertension, and a strategy of comprehensive management of CV risks, including dyslipidemia, impaired glucose tolerance/diabetes or overweight and obesity is highly recommended.

## Conclusions

China has never stopped exploring on the road of prevention and treatment of hypertension. Despite the fact that active and flexible healthcare policies have been made for publics, we have to face the great challenge in diminishing and eliminating the imbalance in economic development in eastern and western region of China. Guideline-recommended five classes of BP-lowering medications, especially long-acting preparations are not always available or affordable in primary care settings of different areas [15]. BP are uncontrolled currently in over 80% hypertensive population [5]. On the other hand, we have to face the rapid growing of prehypertension, hypertension and CV diseases in younger population in China [16]. There are unmet needs for providing healthcare professionals with a tailored hypertension management strategy in this special population. An expert consensus has been developed and published recently to meet the unmet needs [17].

Based on clinical evidence and expertise, the newest guidelines and consensus will be of help in guiding physicians and practitioners to provide better management of hypertension in China. But we should clearly recognize that there is still a long way to go before the satisfied BP control is achieved.

## Data Availability

Not applicable.

## Abbreviations

ABPM: Ambulatory blood pressure monitoring
ACC: American College of Cardiology
ACEI: Angiotensin-converting enzyme inhibitor
AHA: American Heart Association
ARB: Angiotensin II receptor blocker
BP: Blood pressure
CAD: Coronary artery disease
CCB: Calcium channel blocker
CHL: Chinese Hypertension League
CKD: Chronic kidney disease
CSC: Chinese Society of Cardiology
CSPPT: Chinese Stroke Primary Prevention Trial
CV: Cardiovascular
DBP: Diastolic blood pressure
DM: Diabetes mellitus
ESC: European Society of Cardiology
ESH: European Society of Hypertension
HBPM: Home blood pressure monitoring
HFrEF: Heart failure with reduced ejection fraction
ISH: Isolated systolic hypertension
JSH: Japanese Society of Hypertension
KSH: Korean Society of Hypertension
OBP: Office blood pressure.
NA: Not available
SBP: Systolic blood pressure
SPC: Single pill combination
SPRINT: Systolic Pressure Intervention Trial
TOD: Target organ damage
US: United States

## References

[1] Joint Committee for guideline revision (2019). 2018 Chinese guidelines for prevention and treatment of hypertension—a report of the revision committee of Chinese guidelines for prevention and treatment of hypertension. J Geriatr Cardiol.

[2] Whelton PK, Carey RM, Aronow WS (2018). 2017 ACC/AHA/AAPA/ABC/ACPM/AGS/APhA/ASH/ASPC/NMA/PCNA guidelines for the prevention, detection, evaluation, and Management of High Blood Pressure in adults: a report of the American College of Cardiology/American Heart Association task force on clinical practice Guideliness. Hypertension.

[3] Williams B, Mancia G, Spiering W (2018). 2018 ESC/ESH guidelines for the management of arterial hypertension. Eur Heart J.

[4] Zhou M, Wang H, Zeng X (2019). Mortality, morbidity, and risk factors in China and its provinces, 1990–2017: a systematic analysis for the global burden of disease study 2017. Lancet.

[5] Wang Z, Chen Z, Zhang L (2018). Status of hypertension in China: results of China hypertension survey, 2012-2015. Circulation.

[6] Wang Z, Hao G, Wang X (2019). Clinical outcomes and economic impact of 2017 AHA/ACC guidelines on hypertension in China. J Clin Hypertens.

[7] Kim H, Ihm S, Kim G (2019). 2018 Korean Society of Hypertension Guidelines for the management of hypertension: part I-epidemiology of hypertension. Clin Hypertens.

[8] Lee H, Shin J, Kim G (2019). 2018 Korean Society of Hypertension Guidelines for the management of hypertension: part II-diagnosis and treatment of hypertension. Clin Hypertens.

[9] Kim K, Ihm S, Kim G (2019). 2018 Korean society of hypertension guidelines for the management of hypertension: part III-hypertension in special situations. Clin Hypertens.

[10] Umemura S, Arima H, Arima S (2019). The Japanese Society of Hypertension Guidelines for the management of hypertension (JSH 2019). Hypertens Res.

[11] Han L, Wu Q, Wang C (2015). Homocysteine, ischemic stroke, and coronary heart disease in hypertensive patients. A population-based, prospective cohort study. Stroke.

[12] Huo Y, Li J, Qin X (2015). Efficacy of folic acid therapy in primary prevention of stroke among adults with hypertension in China: the CSPPT randomized clinical trial. JAMA.

[13] The SPRINT Research Group (2015). A randomized trial of intensive versus standard blood-pressure control. N Engl J Med.

[14] Xie X, Atkins E, Lv J (2016). Effects of intensive blood pressure lowering on cardiovascular and renal outcomes, updated sytemic review and meta-analysis. Lancet.

[15] Su M, Zhang Q, Bai X (2017). Availability, cost, and prescription patterns of antihypertensive medications in primary health care in China: a nationwide cross-sectional survey. Lancet.

[16] Qi Y, Han X, Zhao D (2018). Long-term cardiovascular risk associated with stage 1 hypertension defined by the 2017 ACC/AHA hypertension guideline. J Am Coll Cardiol.

[17] Liu J, Lu X, Chen L, Huo Y (2019). Expert consensus on the management of hypertension in the young and middle-aged Chinese population. Int J Clin Pract.

